# Cardioprotective Strategies from Cardiotoxicity in Cancer Patients: A Comprehensive Review

**DOI:** 10.3390/jcdd9080259

**Published:** 2022-08-11

**Authors:** Christos Kourek, Maria Touloupaki, Athanasios Rempakos, Konstantinos Loritis, Elias Tsougkos, Ioannis Paraskevaidis, Alexandros Briasoulis

**Affiliations:** 1 Medical School of Athens, National and Kapodistrian University of Athens, 11527 Athens, Greece; 2 Department of Cardiology, Hygeia Hospital, 15123 Athens, Greece; 3 Division of Cardiovascular Medicine, Section of Heart Failure and Transplantation, University of Iowa, Iowa City, IA 52242, USA

**Keywords:** cardiotoxicity, cancer patients, chemotherapy, cardioprotective strategies, risk factors, medical therapy

## Abstract

Cardiotoxicity is a significant complication of chemotherapeutic agents in cancer patients. Cardiovascular incidents including LV dysfunction, heart failure (HF), severe arrhythmias, arterial hypertension, and death are associated with high morbidity and mortality. Risk stratification of cancer patients prior to initiation of chemotherapy is crucial, especially in high-risk patients for cardiotoxicity. The early identification and management of potential risk factors for cardiovascular side effects seems to contribute to the prevention or minimization of cardiotoxicity. Screening of cancer patients includes biomarkers such as cTnI and natriuretic peptide and imaging measurements such as LV function, global longitudinal strain, and cardiac MRI. Cardioprotective strategies have been investigated over the last two decades. These strategies for either primary or secondary prevention include medical therapy such as ACE inhibitors, ARBs, b-blockers, aldosterone antagonists, statins and dexrazoxane, physical therapy, and reduction of chemotherapeutic dosages. However, data regarding dosages, duration of medical therapy, and potential interactions with chemotherapeutic agents are still limited. Collaboration among oncologists, cardiologists, and cardio-oncologists could establish management cardioprotective strategies and approved follow-up protocols in patients with cancer receiving chemotherapy.

## 1. Introduction

Novel cancer therapies have significantly improved survival among patients with malignancies during the last decades. However, they have also resulted in increased morbidity and mortality among cancer patients due to adverse effects. Cardiotoxicity is the most significant complication of chemotherapeutic agents leading, thus, to increased morbidity and mortality, impaired cardiac and endothelial function, and decreased quality of life. Specifically, the prevalence of asymptomatic cardiac dysfunction years after chemotherapy with anthracyclines could even rise up to 57% in cancer patients in Western countries while data in Africa are still limited [1,2]. The prevalence of chronic heart failure secondary to cancer-therapy-related cardiotoxicity is approximately ≈1 million people in Europe and almost 1 in every 20 cancer patients in Asia [2].

Therefore, cardioprotective strategies are of paramount importance. They include primary and secondary prevention. It is crucial to risk stratify cancer patients prior to therapy initiation to recognize those at high risk for cardiotoxicity and follow them up throughout and after the therapeutic process in order to treat in a timely manner for cardiovascular therapy effects.

Current therapeutic protocols often include multiple agents resulting in additive or synergistic cardiotoxic effects. Cancer therapeutic agents that are mainly linked to cardiovascular toxicity include anthracyclines, human epidermal growth factor-2 inhibitors (HER2s), vascular endothelial growth factor inhibitors (VEGFs), Bcr-Abl kinase inhibitors (Bcr-Abls), proteasome inhibitors (proteasomes), ICs, and ibrutinib. Radiotherapy and hormone therapy effects are out of the purpose of this article.

The aim of this review is to demonstrate the most updated cardiopreventive/cardioprotective strategies for patients with cancer with clinical left ventricular (LV) dysfunction induced by cardiotoxicity and in asymptomatic patients with subclinical LV dysfunction.

## 2. Preventive Strategies

The appropriate selection of cancer patients who could benefit from cardioprotective strategies still remains a major issue. There is a lack of agreement on the definition of high-risk patients and limited data to support specific preventive strategies in certain patient populations. Most trials focus on systolic dysfunction and biomarkers.

Interestingly, there is no universally accepted definition for cardiac toxicity. The Cardiac Review and Evaluation Committee in an attempt to combine different definitions from various organizations proposed the presence of at least one of the following criteria for the diagnosis of cardiotoxicity: (1) cardiomyopathy characterized by a decrease in cardiac LVEF, either global or more severe in the interventricular septum, (2) symptoms of congestive heart failure (CHF), (3) associated signs of CHF, including but not limited to S3 gallop, tachycardia, or both, and (4) decrease in LVEF of at least 5% to less than 55% with accompanying signs or symptoms of CHF, or a decline in LVEF of at least 10% to below 55% without accompanying signs or symptoms [1].

Cardiovascular complications encompass variable entities apart from myocardial dysfunction and heart failure (HF), such as valvular disease, pulmonary hypertension, pericardial complications, coronary artery disease (CAD), arrhythmias, arterial hypertension, thromboembolic disease, peripheral vascular disease, and stroke. Cardiotoxic effects can occur either in the short or in the long-term following treatment and they may be transient or irreversible [2].

## 3. Primary Prevention of Cardiotoxicity

### 3.1. Identification and Management of Cardiotoxicity Risk Factors

**Figure 1:**
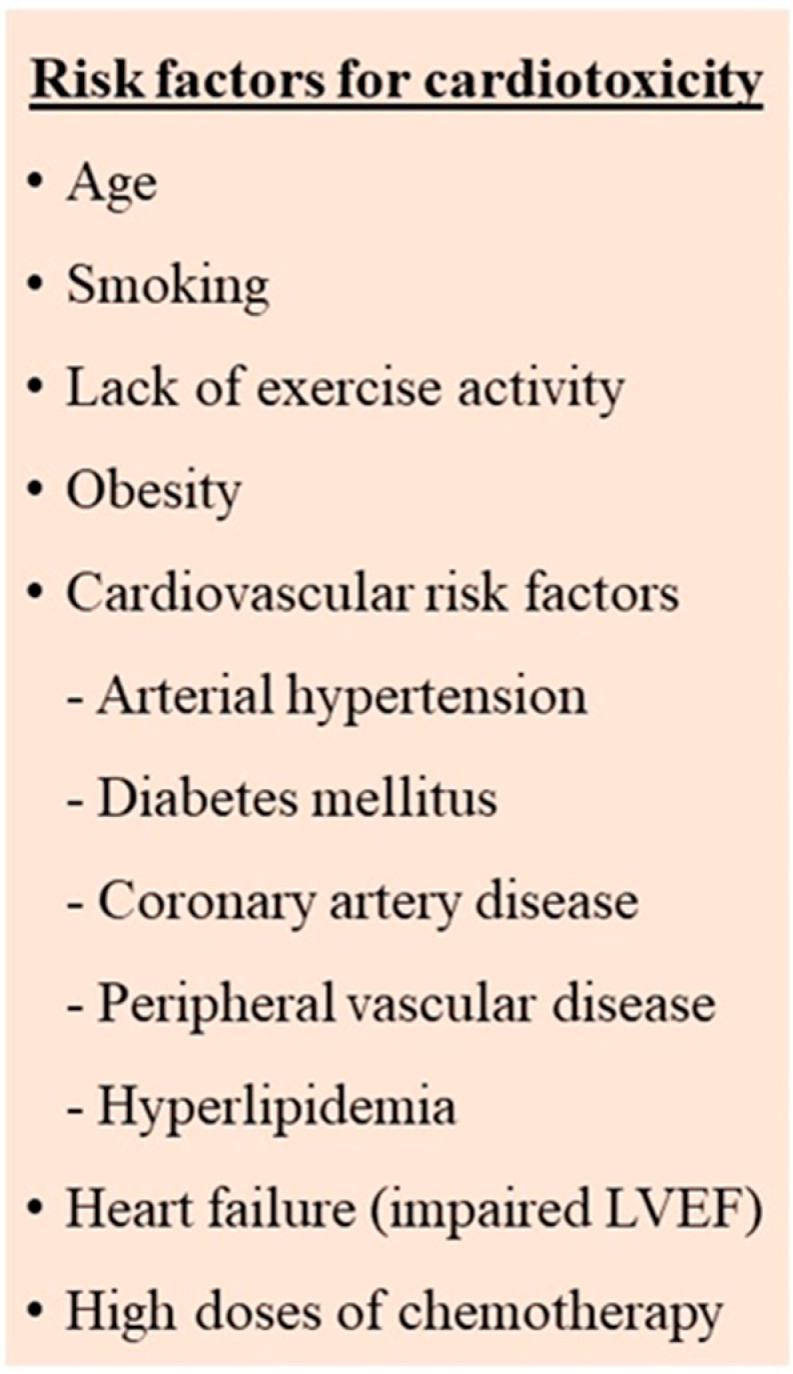
Risk factors for cardiotoxicity in cancer patients receiving chemotherapy.

The early identification and management of the risk factors for cardiovascular side effects seems to contribute to the prevention or minimization of cardiotoxicity. Numerous risk factors, both patient-related as well as therapy-related, have been described (Figure 1) [2,3,4,5]. However, there are still differences in the definition of the high-risk patient as well as the type and the timing of the recommended investigations.

The main patient-related risk factors appear to be the pre-existence of cardiac risk factors such as diabetes mellitus, hypertension, dyslipidemia, smoking, increased body weight as well as previous history of cardiovascular disease, left ventricular dysfunction, heart failure, and coronary artery disease. Other factors such as chronic kidney disease, increasing age, female gender, and postmenopausal status have also been proposed. Active management of the modifiable risk factors according to current guidelines is needed. Additionally, tobacco cessation, regular exercise, and a healthy diet are recommended as primary preventive measures to improve outcomes [2,3,5,6].

At the moment, there are no specific cardiovascular risk scores for cancer patients that can accurately calculate their risk. Therefore, the assessment of these patients using the risk scores for the general population is recommended at the time of diagnosis [7].

A retrospective cohort study in 36,232 adult cancer patients ≥2-year survivors, showed that survivors with two or more cardiovascular risk factors had the highest percentage of ischemic heart disease, stroke, and cardiomyopathy, heart failure when compared to noncancer matched controls. Overall survival in cancer patients who developed cardiovascular disease (CVD) was poor, accounting for 60% at 8 years compared to 81% in cancer survivors without CVD, underlying the need for cardioprevention in individuals at highest risk for cardiovascular disease [8].

The principal therapy-related factors include the combination of multiple agents, (particularly if they are administered simultaneously or in bolus doses), the addition of mediastinal radiotherapy, and higher doses of chemotherapeutic agents.

Certain agents, such as anthracycline, trastuzumab, and cyclophosphamide give a higher cardiotoxicity risk while others such as etoposide, bevacizumab, and lapatinib seem to carry a lower risk [9]. Of note, previous anthracycline treatment increases the risk in patients presenting with recurrent disease or even a new malignancy requiring further anthracycline therapy [10]. Genetic polymorphisms have also been described as possible predisposing factors at lower anthracycline doses, suggesting that the genetic substrate could modify the risk of cardiotoxicity after cancer treatment [2,11].

### 3.2. Cardiovascular Assessment

A thorough cardiovascular assessment and active surveillance are needed before, during, and following the therapeutic process in order to prevent and/or detect cardiovascular toxicity. A combination of imaging evaluation and serum biomarkers has been suggested by several Cardiology and Oncology societies [12,13,14,15].

The French Working Group of Cardio-Oncology proposes the combination of the following cardiotoxicity risk factors, taking into account the patient’s previous history, the biomarkers, the dose and type of the therapeutic agents, and imaging findings: previous heart disease, elevated cardiac biomarkers before initiation of anticancer therapy (N-terminal pro-B-type natriuretic peptide or B-type natriuretic peptide and/or troponin), high-dose anthracycline therapy (e.g., doxorubicin ≥ 250 mg/m^2^, epirubicin ≥ 600 mg/m^2^), high radiotherapy dose (≥30 Gy) with the heart in the treatment field or lower-dose of anthracycline or HERs or VEGFs or proteasomes or Bcr-Abls but with the presence of any of the following risk factors: older age ≥ 60 years, lower radiotherapy dose (<30 Gy) where the heart is in the treatment field, ≥2 risk factors which include diabetes mellitus, hypertension, dyslipidemia, smoking, chronic kidney disease, and obesity [3].

#### 3.2.1. Biomarkers

Elevated serum cardiac biomarkers before therapy initiation have been associated with a higher cardiotoxicity risk. They can be used to identify subclinical cardiac damage. However, there are inadequate data on the subgroups of patients and the time intervals they should be measured.

Cardiac troponin is considered a predictor for LV dysfunction in patients receiving chemotherapy, especially with certain agents such as anthracyclines. However, this is based on studies with small sample sizes, heterogeneous populations, and non-standardized intervals of troponin measurement. It is recommended troponin levels be tested only in high-risk patients.

Increased troponin I (cTnI) values during high-dose chemotherapy have been an established strong predictor of future cardiac dysfunction [16]. In a multicenter study with a small sample size of 78 patients with breast cancer undergoing doxorubicin and trastuzumab therapy, an early increase in ultrasensitive troponin I (TnI) and myeloperoxidase (MPO) levels offered additive information on the risk of cardiotoxicity. TnI was associated with subsequent cardiac dysfunction and heart failure in these patients and MPO seemed to be a potential marker of cardiac dysfunction [15]. In an unselected group of 555 cancer patients, the elevated levels of N-terminal pro-brain natriuretic peptide, mid-regional pro-atrial natriuretic peptide, mid-regional pro-adrenomedullin, high-sensitivity troponin T and copeptin prior to anticancer therapy were strongly associated with all-cause mortality [13].

A multi-marker approach or the combined use of global longitudinal strain in transthoracic echocardiography with troponin could improve the prediction of cardiotoxicity [15,17]. Moreover, troponin increases during chemotherapy have been identified as a marker of high-risk patients for initiation of cardioprotective therapy [18]. Of the 473 cancer patients on high-dose chemotherapy participating in this study, 114 (24%) showed elevated troponin I and were randomized to receive or not to receive enalapril. The treatment was started one month after the completion of chemotherapy and continued for 12 months. Left ventricular ejection fraction (LVEF) was similar between groups at the time of randomization. At 1 year, 43% of the control group compared with 0% of the enalapril group met the primary endpoint of an LVEF decrease of >10% from baseline to <50%. Subjects with sustained troponin elevation had greater LVEF reduction [18].

#### 3.2.2. Imaging Techniques

Apart from the left ventricular ejection fraction (LVEF) which has been widely used for the initial assessment and the follow-up of these patients, a complete cardio-oncological evaluation is recommended in the majority of the patients. However, the exact indications, the population who is expected to benefit the most, the timing and the type of examinations need further clarification.

A cardio-oncological evaluation could include clinical examination, electrocardiogram (ECG), blood glucose, lipid profile, glomerular filtration rate calculation, cardiovascular risk assessment, and transthoracic echocardiogram. The role of echocardiography in this setting includes the initial assessment of left ventricular function and the early detection of cardiotoxicity. Left ventricular ejection fraction is the most studied parameter used to assess ventricular function. The echocardiographic study should include measurements of LVEF (ideally three-dimensional but at least two-dimensional Simpson’s biplane method) and global longitudinal strain (GLS). Myocardial strain is considered a useful tool for the detection of cardiotoxicity in the early stages [13].

In the absence of GLS quantification of LV longitudinal function, the use of mitral annular displacement by M-mode echocardiography and/or peak systolic velocity of the mitral annulus by pulsed-wave DTI could be used. Left ventricular contrast agents could be potentially useful in two-dimensional echocardiography [3]. Of note, the combination of cardiac troponin levels and longitudinal strain could predict the occurrence of cardiotoxicity in patients receiving chemotherapy with anthracyclines and trastuzumab [16].

Cardiac echo with 3D LVEF assessment is another significant tool that has the possibility to identify asymptomatic subclinical LV dysfunction with increased sensitivity in patients with malignancies undergoing chemotherapies [1]. Indications for the use of 3D echocardiography for the assessment of cancer patients before chemotherapy are the same as in 2D echocardiography. The purpose of the use of 3D echo is the early detection of subclinical cardiotoxicity in patients who are treated with anthracyclines in order to decrease the risk of progression to HF, and thus, improve the quality of life and outcomes in these patients [2].

The most specialized and detailed method for LVEF assessment is cardiac MRI. However, is it used only in cases where image quality is low and unclear with echo or other imaging techniques. Cardiac MRI and multigated acquisition angiograms are being used only as alternatives due to the high cost which is prohibitive for screening of cancer patients [12,19].

**Figure 2:**
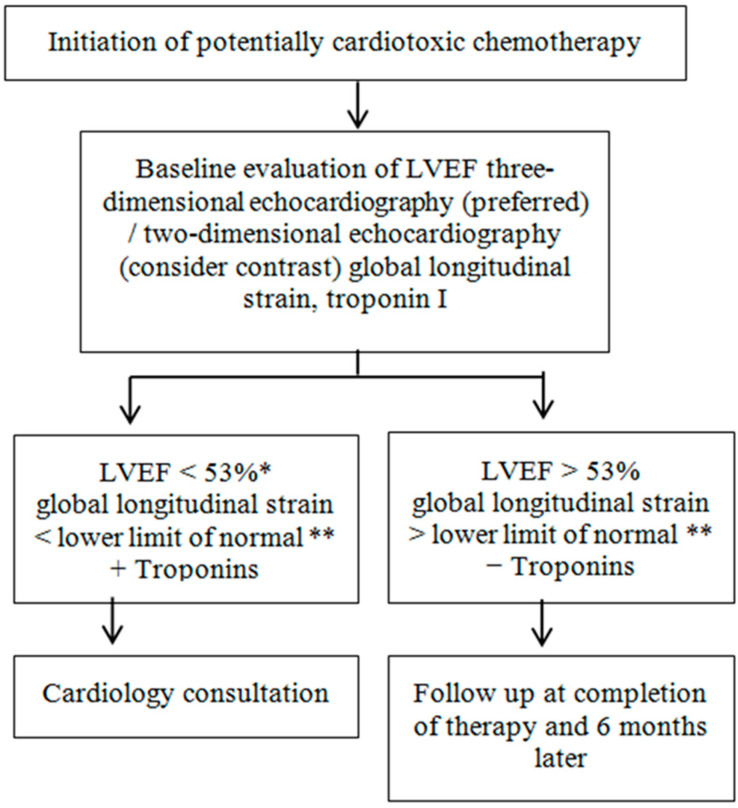
Evaluation of echocardiographic indices and biomarkers in cancer patients prior to the initiation of chemotherapy and proposed follow-up protocols. * Consider confirmation with cardiac magnetic resonance. ** Refer to the American Society of Echocardiography guideline for normal global longitudinal strain values based on vendor, gender, and age. Modified from Expert Consensus for Multi-Modality Imaging Evaluation of Adult Patients During and After Cancer Therapy.

LVEF function assessment should be repeated twice in patients who are going to receive trastuzumab after the use of an anthracycline; once after completion of the anthracycline and once prior to initiation of trastuzumab. Cardiac function assessment could be suggested at 3, 6, 9, and 12 months after the use of an anthracycline or analogs, while in patients with metastatic disease only at baseline and then in the presence of HF or other significant cardiovascular complications [19]. A proposed follow-up algorithm by the American Society of Echocardiography is presented in Figure 2.

### 3.3. Cardioprotective Medical Therapy

**Figure 3:**
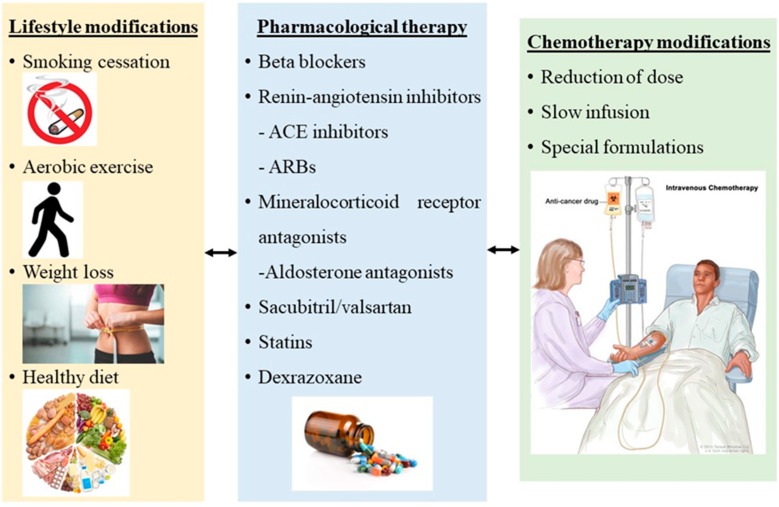
Cardioprotective strategies against chemotherapy cardiotoxicity in cancer patients.

During the last two decades, the established and most approved strategic management of prevention and treatment of chemotherapy-induced cardiotoxicity in HF with LV dysfunction and LVEF < 40% is the use of b-blockers, renin–angiotensin inhibitors including angiotensin-converting enzyme (ACE) inhibitors and angiotensin receptor blockers (ARBs), statins, the use of dexrazoxane and a non-pharmaceutical approach, physical exercise (Figure 3) [19].

Beta-blockers have the ability to increase prosurvival signaling through the EGFR pathway and mitigate free radicals. Several b-blockers have been used in several studies within the last years [20,21,22,23,24,25,26,27,28], but carvedilol and nebivolol seem to be the most efficient so far. Specifically, carvedilol is a third-generation nonselective BB that reduces free radicals, prevents mitochondrial dysfunction, and inhibits lipid peroxidation [29,30]. Daily use of carvedilol twice a day has been shown to contribute to lower troponin I levels and lower incidence of diastolic dysfunction in patients with cancer under chemotherapeutic agents compared to controls [20,23,26,28], as well as unchanged dimensions in LV basal septal, lateral peak systolic strain and strain-rate parameters after chemotherapy compared to controls [21,22]. Another b-blocker, nebivolol is another third-generation BB with vasodilatory and antioxidant properties that increases nitrous oxide and decrease reactive oxygen species [31]. It has been shown to have similar beneficial effects in myocardium as 5 mg on a daily basis protects from impairment in LVEF compared to controls [25].

Another category of cardioprotective medication for cardiotoxicity is renin–angiotensin inhibitors including ACE inhibitors and ARBs. ACE inhibitors and ARBs are neurohormonal blocking agents, used to treat hypertension and facilitate cardiac remodeling [32]. Their action is through the attenuation of oxidative stress and myocardial fibrosis [18,33]. They also improve intracellular calcium handling, cardiomyocyte metabolism, and mitochondrial function [18,33]. Lisinopril has been shown to protect LVEF and present lower long-term cardiovascular events compared to the placebo group, in a study of 114 patients receiving anthracyclines [18]. Enalapril is another ACE inhibitor that has been used daily as a protective strategy in patients receiving chemotherapeutic agents. In a study by Cardinale et al. [34], daily use of enalapril at the start of chemotherapy showed significantly lower elevation of troponin and fewer cardiotoxicity incidents compared to the troponin-triggered enalapril therapy group. Another study by Janbabai et al. [35], showed that daily use of enalapril results in lower incidences of LV diastolic dysfunction from baseline at 6 months, and significantly unchanged tissue Doppler, E/e’ ratio, mean LVEF, and cTnI and CK-MB levels compared to the control group where measurements were worse after chemotherapy. An ARB agent, valsartan was also shown to have cardioprotective properties. Specifically, a low dose of 80 mg daily was observed to significantly inhibit the dilatation of LVDd, the elevation of BNP, and the prolongation of the QTc interval and QTc dispersion in 40 patients undergoing chemotherapy [36].

The combination of a b-blockers and a renin–angiotensin inhibitor, however, seems controversial as if it could be suggested as the most appropriate and efficient method of cardioprotection in patients with malignancies undergoing chemotherapy. Two big studies, the OVERCOME and the PRADA trial, combined a b-blocker and a renin–angiotensin inhibitor in the intervention group and compared it to a control group of either placebo treatment or treatment with a b-blocker or a renin–angiotensin inhibitor separately. In the first trial [37], investigators administered carvedilol and lisinopril together at the start of chemotherapy to their patients and compared it to a control group of placebo treatment. The combination was shown to be effective at preventing the decline in LVEF compared to the placebo. In the other trial, the PRADA trial [38], investigators examined the combination of 32 mg of candesartan and 100 mg of metoprolol versus candesartan alone, or metoprolol alone or placebo therapy. They observed that breast cancer patients receiving canderstartan during anthracycline chemotherapy had less LVEF decline but metoprolol did not have the same effect. They also observed that there was no additional benefit when metoprolol was used in conjunction with candesartan [38]. Finally, another big trial, the MANTICORE 101–Breast trial [39], investigated the early treatment with perindopril, bisoprolol, or placebo (1:1:1) in patients with HER2-positive early breast cancer for the duration of trastuzumab adjuvant therapy. It was shown that perindopril and bisoprolol protected against cancer-therapy-related declines in LVEF while LV remodeling could not be prevented. There were no other studies demonstrating the beneficial effects of the combination of b-blockers and renin–angiotensin inhibitors.

The use of another medication, sacubitril/valsartan, is still under investigation regarding the cardioprotective effect in cancer patients. Sacubitril/valsartan belongs to the angiotensin receptor-neprilysin inhibitors (ARNIs) and has been approved as a first-line treatment in HFrEF patients. Unfortunately, data are still limited regarding this category. There is a randomized prospective study [40] comparing valsartan/sacubitril to candesartan in 112 breast cancer patients with reduced LVEF prior to getting anthracyclines. This study showed that valsartan/sacubitril group presented less rise in BNP, increased 6-min walk test, better suppression of ventricular arrhythmias, and improved indicators of LV function compared to the candesartan group [40]. However, more randomized clinical trials are required in order to support the use of ARNIs in chemotherapy-related cardiomyopathies.

Mineralocorticoid receptor antagonist blockades, such as aldosterone antagonists, are widely used in patients with HFrEF and suppress fibrosis, leading to improvement of their symptoms [41]. There is a single randomized, double-blind, placebo-controlled study [42] including 43 breast cancer women who received 25 mg of spironolactone daily and were compared to 40 women under placebo treatment. Both groups were receiving doxorubicin or epirubicin. The study showed that spironolactone provided significant short-term cardioprotection by significantly less decrease in LVEF, preserved LV diastolic functional grade, and unchanged serum cardiac biomarker concentrations including creatine kinase-MB, cTnI, and NT-proBNP compared to controls where all indices deteriorated after chemotherapy. Moreover, total oxidative capacity and the oxidative stress index were more pronounced in controls [42].

Anthracyclines increase the reactive oxygen species, oxidative stress and inflammation and cause cardiotoxicity [43]. Statins reduce cholesterol synthesis by inhibiting the enzyme HMG CoA reductase and they are known to exhibit pleiotropic properties and decrease oxidative stress and inflammation [44]. They can also improve endothelial function and nitric oxide delivery [45]. Thus, they may potentially protect against anthracycline-induced cardiac damage [44]. Statins have been used as a cardioprotective strategy against cardiotoxicity in patients with cancer. Specifically, 67 women with newly diagnosed breast cancer were treated with statins during chemotherapy with anthracyclines and had a lower risk of HF (HR, 0.3; 95% CI, 0.1 to 0.9; *p* = 0.03) compared to 134 women not treated with statins [46]. Statins are also shown to prevent a drop in LVEF after chemotherapy with anthracyclines compared to placebo [47]. Indeed, higher statin doses (40–80 mg) could actually have an increase in LVEF [47]. The cardioprotective effects of statins have been also shown in other studies [48,49], not only in anthracyclines but also in trastuzumab therapy [50].

Dexrazoxane is the only approved drug by FDA for preventing anthracycline-induced cardiotoxicity [51]. It has been approved to be given to children and adolescents that are likely to be treated with high cumulative doses of anthracyclines (>300 mg/m^2^ of doxorubicin) [52]. Its action mechanism is to reduce ROS formation via the prevention of anthracycline–iron complex formation [19]. Specifically, dexrazoxane has the ability to bind iron before it enters cardiomyocytes, preventing thus, the formation of the iron–anthracycline complex, free radical formation, and cardiac damage [53,54]. In addition, it prevents anthracyclines from binding to topoisomerase 2β which would lead to cardiomyocyte death and mitochondrial dysfunction [55]. It has been used as a cardioprotective agent against anthracycline-induced cardiotoxicity for over 30 years in many types of solid and hematological malignancies, not only in adults but also in children receiving doxorubicin and other anthracycline drugs. Dexrazoxane has been shown to have fewer rates of asymptomatic LV dysfunction, lower rates of HF progression, better LV performance, and fewer cardiac events in patients treated with doxorubicin and dexrazoxane [56,57,58,59]. Moreover, no changes in cardiac troponin I or brain natriuretic peptide concentrations are observed with dexrazoxane [60]. In summary, dexrazoxane’s effectiveness in reducing anthracycline-related cardiotoxicity in patients with cancer is already proven throughout the years.

Finally, a non-pharmaceutical cardioprotective strategy in patients with cancer under chemotherapy, exercise, has been also studied in the last years. Exercise decreases ROS formation, improves endothelial function, and decreases intracellular anthracycline levels [19,61,62,63]. It also increases heart tolerance against many cardiotoxic agents and therefore improves several functional, subclinical, and clinical parameters [64]. Cancer patients usually decrease their physical activity from pre- to post-diagnosis and gain approximately 3 kg during chemotherapy [65,66]. As a result, their functional capacity is being deteriorated as shown in peak VO2 during cardiopulmonary exercise testing [67]. The American College of Sports Medicine published a consensus regarding exercise safety for specific patient groups with malignancies and cancer survivors, confirming exercise’s overall safety and efficacy [68].

In asymptomatic patients with cancer and subclinical LV dysfunction, these therapies are not usually offered, especially in those with baseline LVEF ≥ 50% [3,5]. The administration of high-dose therapy with anthracyclines is the only case where cardioprotective strategies are being considered. Moreover, in patients with cardiovascular risk factors such as hypertension and/or diabetes, individualized decisions for each patient are being made by the experts. Risk stratification using biomarkers or echocardiographic data is necessary in order to identify patients who may benefit from a cardioprotective approach. The most commonly used cardioprotective strategies in LVEF ≥ 50% with CV risk factors are optimizing management of hypertension, counseling for smoking cessation, weight loss, and physical activity [3,5]. In the other category of asymptomatic patients with subclinical LV dysfunction and LVEF >40 and <50%, optimization of cardiovascular status including blood pressure control in hypertensive patients and careful monitoring of the LVEF and discontinuation of chemotherapy if the LVEF decreases by more than 10 absolute percentage points from baseline are the suggested cardioprotective strategies [3,5]. Initiation and titration of an ACE inhibitor or an ARB plus a beta-blocker prior to starting anthracycline therapy is also significant for this subgroup [3,5].

## 4. Secondary Prevention of Cardiotoxicity

Secondary prevention could be defined as the appropriate management strategies for preventing symptoms, heart failure, and cardiovascular events in asymptomatic anthracycline cardiotoxicity. Although there are no evidence-based guidelines for monitoring cardiotoxicity during and after chemotherapy, there are, however, serum and imaging biomarkers such as cTnI concentration and LVEF in order to detect subclinical cardiotoxicity prior to the development of overt cardiac dysfunction.

Early impaired LV function with LVEF < 40% and HF could be treated with ACE inhibitors in combination with b-blockers if there is no absolute contraindication [69]. B-blockers, and especially metoprolol as a secondary cardioprotection strategy, have been shown to improve LVEF 8 months after the initiation of treatment by 13% in adults with anthracycline-induced cardiomyopathy and LVEF ≤ 45% compared to age-and-sex-matched controls with idiopathic dilated cardiomyopathy under b-blockers [70]. Another medication for cardiology patients, enalapril, has been also examined for secondary prevention of cardiotoxicity, except for primary prevention, in cancer patients. Enalapril was found to protect from myocardial injury, as troponin concentrations did not increase during chemotherapy after its administration to these patients [34]. In another RCT including 114 cancer patients with elevated serum cTnI concentrations, 20 mg/day of enalapril for 1 month after high-dose chemotherapy was shown to prevent cardiotoxicity by preserving LVEF 12 months after chemotherapy compared to cancer patients not receiving medication [18]. Moreover, the cumulative number of adverse cardiac events such as sudden death, death from a cardiac cause, acute pulmonary edema, overt HF, and life-threatening arrhythmias requiring treatment was significantly lower than controls [18]. Finally, a prospective study of 201 consecutive patients with anthracycline-induced cardiomyopathy with LVEF ≤ 45% and HF showed that the combination of enalapril and carvedilol was associated with better LVEF recovery and fewer cardiac events during a follow-up period of 36 months [71]. Thus, enalapril could be recommended as a secondary prevention strategy. The potential mechanisms of ACE inhibitors and b-blockers in cardioprotection are not well established yet, but hemodynamic effects and afterload reduction could explain their effects in preventing cardiotoxicity [44]. An important fact is that ACE inhibitors, ARBs, and b-blockers cannot be used for a long time in cancer patients. B-blockers’ signaling mechanisms and their proangiogenic activity may affect the prognosis of patients with solid cancers, in whom this signaling may facilitate tumor angiogenesis [72]. ACE inhibitors and ARBs might be carcinogenic as both increase the risk of cancer when compared to the placebo [73,74,75]. Finally, another category of medication, aldosterone antagonists, have an increased risk of kidney dysfunction and electrolyte imbalance in the setting of malignancy and chemotherapy [44].

Data regarding prophylactic implantation of cardioverter-defibrillators for primary prevention of sudden cardiac death in patients receiving chemotherapy are still limited. In one single RCT, cardioverter-defibrillators did not benefit patients with non-ischemic cardiomyopathy with an LVEF ≤ 35% and symptomatic systolic HF [76]. The use of these devices in patients with chemotherapy-induced cardiomyopathy remains controversial. Another single-center study including 18 consecutive patients with anthracycline-induced cardiomyopathy demonstrated improvements in echocardiographic markers and clinical benefits with cardiac resynchronization therapy [76].

Finally, new medications used in HF including SGLT-2 inhibitors, ARNIs, and non-steroid MRAs, as well as new agents such as ATPase activators such as omecamtiv mecarbil, could be used as potential secondary prevention strategies with promising results in the future. However, more RCTs are required.

## 5. Discussion

It is a matter of fact that there are significant limitations in investigating cardioprotective strategies of anthracycline-induced cardiotoxicity [77]. The literature lacks a universally accepted definition of cardiac toxicity and the definition of cardiac dysfunction through LVEF alone is insufficient [77]. Moreover, understanding of the pathophysiologic mechanisms of various chemotherapy agents is still limited. All these factors, in combination with the fact that there are no internationally published guidelines to account for different subsets of patient populations, make the creation of new cardioprotective drugs and the establishment of cardioprotective strategies from cardiotoxicity more difficult. Neurohormonal antagonists have been widely used in adults with HF and, especially in the last two decades, in the fragile population of adult cancer survivors, not only for primary prevention of cardiotoxicity but also for secondary prevention as a response to asymptomatic LV dysfunction without clinical HF [41,78]. However, in most studies, sample sizes are quite small and follow-ups are short, up to 12 months. As a result, data are still limited and cannot be generated in bigger populations. There are also unambitious data from a recent review in cancer patients with longer follow-ups which tend to show that treatment with b-blockers and ACE inhibitors or ARBs does not prevent chemotherapy-induced cardiotoxicity [79].

Another significant issue is the error in the equation of LV dysfunction with myocardial damage. LV dysfunction is usually described as alterations in LV function in most studies. However, LV alterations could occur due to changes in the loading conditions of the heart and, thus, these changes in loading conditions do not always equate to myocardial damage [44]. Biomarkers including cTnI and natriuretic peptide, echocardiography measurements including global longitudinal strain, and cardiac MRI are considered significant methods for screening cancer patients, indicating myocardial damage with high sensitivity [80,81,82,83]. However, there are significant knowledge gaps in the detection and prevention of cardiotoxicity. Optimal screening intervals and duration have not yet been defined and there is still a lack of universal agreement on best practices for screening and surveillance intervals.

There are no established “gold standard” medical therapies for primary and secondary prevention of cardiotoxicity. Moreover, there is a lack of established treatment therapies in cancer patients, as well as a lack of data on the safety and feasibility of rechallenging with chemotherapy once LV dysfunction ensues. Medical therapies, usually used in cardiology patients, including ACE inhibitors, ARBs, b-blockers, aldosterone antagonists, and statins have been under investigation in the last years with promising results [19,20,21,22,23,24,25,26,27,28,29,30,31,32,33,34,35,36,37,38,39,40,41,42,43,44,45,46,47,48,49,50,51,52,53,54,55,56,57,58,59,60]. Some studies suggest that the early introduction of cardioprotective therapy is associated with higher chances of LVEF recovery and fewer clinical cardiac events including, HF, arrhythmias, and death [71]. However, there is not enough evidence for factors such as dosages, duration of medical therapy, and potential interactions with chemotherapeutic agents. Indeed, there is no persuasive evidence that ACE inhibitors, ARBs, aldosterone antagonists, or b-blockers in survivors of adult cancers improve survival or quality of life when used for either primary or secondary prevention [44].

As far as patients are concerned, there is still no universal validated risk prediction model that could be used in order to predict and prevent cardiotoxicity before the initiation of chemotherapy. Most trials include younger and low-risk populations and, as a result, this could lead to probable underestimation of cardiovascular incidents, the endpoint outcome in most studies. Another issue for consideration would be the cost of an unproven, potentially long-term therapy, especially in young [84]. The need for a potentially long-term medication for healthy, asymptomatic survivors could also raise daily adherence issues. Moreover, differences exist in the definition of high-risk patients and the recommended strategies for investigation [84].

Finally, there are limited data on novel approaches for management in cancer survivors after chemotherapeutic agents. A novel approach for this specific population would be the potential role of cardiac progenitor cells in the therapeutic strategies or the potential role of genetic polymorphisms in cardiotoxicity [85,86,87]. However, this field remains understudied and further investigation is required.

## 6. Limitations of Studies on Cardioprotective Strategies

There are limitations in studies regarding cardioprotective strategies in cancer patients receiving chemotherapy. Firstly, most studies include small sample sizes due to the fact that cardio-oncology still lacks evidence and remains an understudied field in medicine. The heterogeneity of samples makes the investigation of cardioprotective strategies more difficult. In addition, there are low event rates and short follow-up durations in these patients, probably because most malignancies may be aggressive with a low 1- or 5-year survival or a high percentage of patients may be end-stage. The lack of established serum markers or echocardiography measurements demonstrating anthracycline-induced myocardial injury in cancer patients’ follow-up is another significant limitation.

## 7. Conclusions

Cardiotoxicity, induced by anthracyclines and analogs, is a major problem in cancer patients receiving chemotherapy with significant morbidity and mortality. During the last decades, cardioprotective strategies have been proposed in order to prevent or limit the side effects of chemotherapy in these patients. Drug medications, implantation of cardioverter-defibrillators, physical therapy, and reduction of chemotherapy doses are some of these strategies. Serum biomarkers including cTnI concentration or echocardiography measurements including LVEF and global longitudinal strain could be potential follow-up indices, however, are still not widely approved yet. Taking all data from the literature into consideration, it seems that there is no clear consensus concerning recommendations for cardioprotective pharmacotherapy, but a broad agreement regarding the importance of rigorous risk factor control and treatment. Only a collaboration among oncologists, cardiologists, and cardio-oncologists could establish management cardioprotective strategies in patients with cancer receiving chemotherapy. Medication including ACE inhibitors, ARBs, b-blockers, aldosterone antagonists, statins, and dexrazoxane could play a significant role in primary and secondary cardioprotection from anthracycline-induced cardiotoxicity. However, their potential mechanisms remain unknown. More RCTs are required in order to establish evidence-based algorithms and create individualized approaches for patients with cancer.
